# Research Collaborations in the Post-COVID Era

**DOI:** 10.31662/jmaj.2023-0034

**Published:** 2023-05-12

**Authors:** Takahiro Kaneko, Soichiro Saeki

**Affiliations:** 1 Center Hospital of the National Center for Global Health and Medicine, Tokyo, Japan

**Keywords:** Communication, Academic Meeting, Internet of Things, Information Systems, Japan

The COVID-19 pandemic has drastically transformed communication methods. Face-to-face interactions became extremely limited, forcing students to miss once-in-a-lifetime opportunities ^(1)^. Similarly, researchers have resorted to virtual conferences and meetings ^(2)^. This shift has fueled technological advancements and made individuals more proficient in virtual environments ^(3)^.

As health restrictions are gradually relaxed, hybrid meetings that combine in-person and virtual attendance are becoming increasingly common ^(2)^. These gatherings were useful because they eliminated geographical and administrative barriers ^(4)^. Moreover, many researchers currently prefer hybrid meetings to other platforms, and they are expected to become the new norm after the pandemic ^(5)^. Researchers may prefer to attend meetings virtually rather than in person, because virtual attendance is more convenient and efficient than traveling to a conference venue ^(2), (4)^. Although hybrid meetings have numerous benefits, face-to-face meetings have two distinct advantages.

First, in-person communication is easier and more casually initiated as people are in close proximity. Face-to-face communication allows for an unplanned conversation with someone you did not intend to speak with and discussion of topic you had never considered before. Therefore, informal conversations that occur unintentionally, such as those that occur during meals or social gatherings, can occasionally lead to new ideas and networking opportunities. A conversation with a coworker from another laboratory who is seated next to you could spark new ideas and cross-laboratory collaborations. However, during online conversations, numerous impediments to casual “chatting” exist. Thus, while online meetings are convenient and efficient, in-person interactions are required to spark creativity and establish new relationships.

Second, physical encounters involving multiple senses provide people with new perspectives. For example, traveling to a new location for attending an academic conference or studying at another institution, can teach you about local culture and social structures. Understanding and recognizing cultural differences will broaden one’s horizons as a person and researcher. Grasping the social context of science allows researchers to focus on how their work benefits the society. Thus, online meetings can offer fresh perspectives; however, the information we receive is limited to visual and auditory cues. Furthermore, attending meetings in unfamiliar locations may lead us to step outside our comfort zones.

To summarize, hybrid meetings offer flexibility and ease of communication; however, it is important to strike a balance between the convenience of virtual attendance and the benefits of face-to-face interaction. Hence, in-person meetings should not be neglected in pursuit of convenience and simplicity with hybrid meetings.

## Article Information

### Conflicts of Interest

None

### Acknowledgement

The authors are grateful to their colleagues, specifically those in clinical residency training, for their helpful discussions. The authors are also thankful to Paperpal (Cactus Communications Services Pte Ltd, Singapore) and ChatGPT (OpenAI, L.L.C., San Francisco, CA, USA) for providing primary language editing.

### Author Contributions

Both authors conceptualized the manuscript. KT wrote the original draft and SS critically edited it. Both authors read and approved the final version of the manuscript.

### Approval by Institutional Review Board (IRB)

Not applicable.
